# Identify the Characteristics of Metabolic Syndrome and Non-obese Phenotype: Data Visualization and a Machine Learning Approach

**DOI:** 10.3389/fmed.2021.626580

**Published:** 2021-04-07

**Authors:** Cheng-Sheng Yu, Shy-Shin Chang, Chang-Hsien Lin, Yu-Jiun Lin, Jenny L. Wu, Ray-Jade Chen

**Affiliations:** ^1^Department of Family Medicine, Taipei Medical University Hospital, Taipei, Taiwan; ^2^Department of Family Medicine, School of Medicine, College of Medicine, Taipei Medical University, Taipei, Taiwan; ^3^Graduate Institute of Biomedical Informatics, College of Medical Science and Technology, Taipei Medical University, Taipei, Taiwan; ^4^Professional Master Program in Artificial Intelligence in Medicine, College of Medicine, Taipei Medical University, Taipei, Taiwan; ^5^Division of General Surgery, Department of Surgery, Taipei Medical University Hospital, Taipei, Taiwan; ^6^Department of Surgery, School of Medicine, College of Medicine, Taipei Medical University, Taipei, Taiwan

**Keywords:** machine learning, metabolic syndrome, non-obese phenotype, data visualization, preventive medicine, artificial intelligence

## Abstract

**Introduction:** A third of the world's population is classified as having Metabolic Syndrome (MetS). Traditional diagnostic criteria for MetS are based on three or more of five components. However, the outcomes of patients with different combinations of specific metabolic components are undefined. It is challenging to be discovered and introduce treatment in advance for intervention, since the related research is still insufficient.

**Methods:** This retrospective cohort study attempted to establish a method of visualizing metabolic components by using unsupervised machine learning and treemap technology to discover the relations between predicting factors and different metabolic components. Several supervised machine-learning models were used to explore significant predictors of MetS and to construct a powerful prediction model for preventive medicine.

**Results:** The random forest had the best performance with accuracy and c-statistic of 0.947 and 0.921, respectively, and found that body mass index, glycated hemoglobin, and controlled attenuation parameter (CAP) score were the optimal primary predictors of MetS. In treemap, high triglyceride level plus high fasting blood glucose or large waist circumference group had higher CAP scores (>260) than other groups. Moreover, 32.2% of patients with high CAP scores during 3 years of follow-up had metabolic diseases are observed. This reveals that the CAP score may be used for detecting MetS, especially for the non-obese MetS phenotype.

**Conclusions:** Machine learning and data visualization can illustrate the complicated relationships between metabolic components and potential risk factors for MetS.

## Introduction

Because of the increasing prevalence of obesity, metabolic syndrome (MetS) has become a common metabolic disorder. There are several diagnostic criteria for MetS including National Cholesterol Education Program's Adult Treatment Panel III (ATP III), Modified ATP III for Asians, International Diabetes Federation (IDF) Criteria, National Heart, Lung, and Blood Institute (NHLBI) Criteria, and Joint Interim Statement of the International Diabetes Federation Task Force on Epidemiology and Prevention; National Heart, Lung, and Blood Institute; American Heart Association; World Heart Federation; International Atherosclerosis Society; and International Association for the Study of Obesity (JIS) (1–4). A comparison of the above diagnostic criteria for MetS, which is relevant for Asians can be found in Supplementary Table 1. In general, these different Mets criteria are very similar, all of them looks at the presence of ≥ three anthropometric characteristics or clinical factors as listed below: large waist circumference (WC), high triglyceride level (TG), high blood pressure (BP), high fasting blood glucose (FBG), and low high-density lipoprotein (HDL) cholesterol level. When evaluating Mets for Asians, the modified ATP III, JIS, and NHLBI criteria are almost identical. The IDF criteria are the most different from the above three criteria as the criteria insist that a Mets person must have abdominal obesity.

In previous studies, Beydoun et al. assessed the adiposity indices for MetS from a cohort data, the performance of detecting MetS was 0.680 and 0.733 for men using body fat mass and WC, respectively, and women (0.581 vs. 0.686) (5). Zhang et al. used a routine biomarker-based risk in Cox regression to predict MetS in an urban Han Chinese population, the performance was 0.796 and 0.897 for males and females (6). Both studies only had a better performance on females, and the selection of predictors is not objective and automated.

Non-alcoholic fatty liver disease (NAFLD) is a common comorbidity that is correlated with overweight and MetS. NAFLD is now primarily considered as a hepatic manifestation of MetS. Nevertheless, plenty of research has shown that NAFLD affects not only the liver but other chronic diseases such as chronic kidney disease (CKD), type 2 diabetes mellitus, and cardiovascular disease. Therefore, many chronic MetS-related diseases are directly caused by NAFLD, and better diagnoses and therapies of fatty liver disease are highly necessary (7–11). Currently, the detection of NAFLD has been enhanced with the capability of quantifying hepatic steatosis via measuring ultrasonic attenuation at the central frequency of the Fibroscan, termed the controlled attenuation parameter (CAP) (12–14). Previous study has found that CAP score alone can detect Mets with reasonable high accuracy of 0.79 and the combined use with machine learning can improve Mets accuracy detection to 0.904 (15, 16).

Machine learning is an artificial intelligence technique in which can the algorithm automatically learns and improves from experience or large amounts of data without being explicitly programmed. The kernel of machine learning is a statistical analysis that provides a powerful and purposeful method of observing specific patterns and correlations in health care issues by exploring undiscovered data, resulting in the establishment of data-driven prediction models (16–21). Several clinical issues—such as chronic kidney disease, postoperative sepsis, and alexithymia in fibromyalgia—have been explored using machine learning (22–24).

Data visualization is a useful technique that enhances clinicians' ability to analyze and summarize complex and large volumes of clinical data. Treemap visualization in particular is a conceivably advantageous method of visualizing clinical health care data. It enables the representation of high-dimensional hierarchical data in one diagram (21, 25, 26).

In this study, we will like to combine the use of data visualization and machine learning to find out if different levels of Mets will have different prediction accuracies. This is because the non-obese MetS population is difficult to discover, and this population is always the most challenging target in preventive medicine. In addition, we will like to find out if the CAP score alone can detect non-obese patients, as currently there are limited tools to detect non-obese patients without the invasive blood draw and inconvenient starvation. Use of CAP score for screening offers the clinical advantage of non-invasiveness, and no requirement for overnight starvation.

## Methods

### Setting and Study Design

This retrospective cohort study was executed at Taipei Medical University Hospital (TMUH), a private teaching hospital with 800 beds in Taiwan. The electronic health care records of all participants were analyzed. The ability of treemap visualization and supervised machine learning to cluster different combinations of five metabolic components was assessed using patients who took a self-paid health examination at the Healthcare Center (HC) of TMUH, which has approximately 60 visits per day. This study was approved by the Institutional Review Board of TMUH (TMU-JIRB No.: N202003088).

### Data Collection and Criteria

Patients had to meet the following inclusion criteria: older than 17 years, underwent a self-paid health examination at the HC of TMUH between March 2015 and May 2019, and underwent abdominal transient electrography inspection using the FibroScan 502 Touch (Echosens, Paris, France).

**Figure 1:**
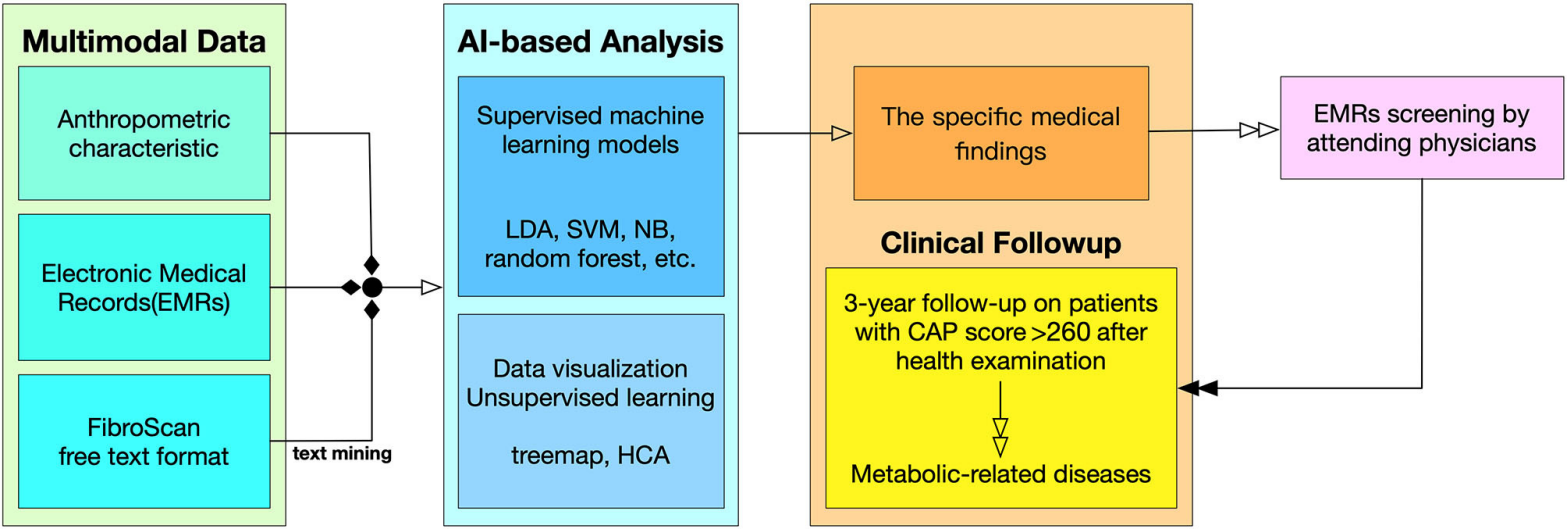
Flowchart from data collection to clinical follow-up. Multimodal Data. The input variables are divided into three types of sources in the clinical data collection. The detail list of inputs can be found in Supplementary Table 3. AI-Based Analysis. This study used both supervised machine learning model and unsupervised machine learning along with data visualization techniques. Clinical Follow-up. The result of the AI-based analysis feedback to physicians, and the physicians investigate the outcome of the patients through EMRs screening.

All patients underwent the regular processes of the HC (Supplementary Table 2). The blood samples required were collected from laboratory tests, and other anthropometric characteristics were also recorded (Supplementary Table 3). The definitions of measurement cut-offs and calculations are presented in Supplementary Tables 1, 4. The included patients were than follow-up for 3 years at Taipei Medical University Hospital (Figure 1), and it was found that ~60% of patients do not have follow-up data.

### Statistical Analysis

The chi-square test and Kruskal–Wallis rank sum test were used to compare the groups of various participants with different numbers of metabolic components. Descriptive characteristics were also analyzed and are presented as discrete or continuous variables with frequencies or percentages and medians or interquartile ranges, respectively. A box plot was drawn for presenting data distributions and comparing groups. Multinomial stepwise logistic regression was used to determine which variables had significant differences and the odds ratios among the groups of patients with different numbers of metabolic components. Receiver operating characteristic (ROC) curves were plotted to demonstrate the diagnostic ability of machine-learning prediction models for MetS. Model performance was measured using c-statistic, sensitivity (recall), and specificity in ROC plots (27, 28).

Figure 1 describes the procedure of this study from data collection to clinical outcomes. In data preprocessing, multimodal data were summarized; a series of machine learning models were then constructed, and statistical analyses were performed. A feedback mechanism was working clinically as a prospective survey when remarkable findings were obtained by the machine learning models. A recommended threshold of risk factor was targeted before clinical physician scrutinized the potential MetS patients' follow up (16).

### Machine Learning

Several supervised machine-learning models—k-nearest neighbor classification (KNN), linear discriminant analysis (LDA), logistic regression for classification, ensemble learning, support vector machine (SVM), naive Bayes classification (NB), and hierarchical clustering analysis (HCA)—were also executed using R (version 3.6.3). The factors used as input to each machine learning models were listed in Supplementary Table 3. And a series of data preprocessing, including structured query language command, database merging and text mining, were applied to integrate these databases in the study.

KNN has relatively simple implementation and is robust because the classes do not have to be linearly separable in the searching space. This advantage was the reason it was applied to missing value mutation in our study (29, 30). Variables will be excluded if the number of missing values is more than 10% of the sample size in this study.

LDA is a statistical method in which a linear combination of features separating two or more classes of objects is located. It can handle multivariate problems because its linear combination is more commonly used for dimensionality reduction before classification (31, 32).

Logistic regression is usually used in machine learning for classification because the probability of some obtained event is represented as a linear function of a combination of predictor variables. The technique is used when the response variable is categorical in nature, for instance, when it has the value yes/no or true/false. In contrast to linear regression, a linear relationship between dependent and independent variables is not required (33).

The main advantage of ensemble models in machine learning is that decisions from multiple models are combined to improve overall performance (34, 35). Random forest is a parallel ensemble method used for classification, regression, or other applications and is based on the structure of a decision tree. It eliminates the possibility of bias that a decision tree model may induce in the system. Moreover, it improves the predictive power considerably (36). Adaptive boosting (AdaBoost) is a sequential ensemble method in which the base learners are generated in series. The underlying purpose of sequential learning is to use the dependence between the base learners, and overall performance can be improved by giving previously mislabeled samples higher weights in the sequential training processes (37, 38).

SVM model constructs a hyperplane or set of hyperplanes in a high-dimensional space, which is used for classification, regression, or outlier detection. Although SVM performs relatively favorably when a clear margin of separation exists between classes, and it is effective in high-dimensional spaces (39).

NB classifiers are probabilistic classifiers based on the use of Bayes' theorem with naive assumptions of independence between features. They are simple and easy to implement and do not require as much training data as other methods. The leading advantage of NB classification is that it is highly scalable with the number of predictors and data points (40).

The machine learning algorithms were executed in R program, the library, package and function using in this study are listed in Supplementary Table 5.

### Data Visualization

In data analysis, visualization is always the most intuitive and sufficient method of exploring a specific pattern in data reflecting unknown or complicated issues. In this study, we used an unsupervised learning model called HCA in heatmap and a large and complex data-mapping technique called treemap to depict the characterization of metabolic components, because these approaches clearly enable recognition of special patterns in high-dimensional data through the use of gradient colors and grids of different areas (26, 41).

## Result

The statistical distribution and differences between patient groups with different numbers of metabolic components are shown in Table 1. The combinations of metabolic components are listed in Supplementary Table 6. Stepwise multinomial logistic regression reveals the odds ratios, compared with the healthy group without any metabolic components, among the significant variables in Table 2. When the number of metabolic components increases, a significant difference was observed in several predictors, such as age, body mass index (BMI), gamma-glutamyl transferase (γGT), CAP score, serum uric acid (UA), cholesterol, low density lipoprotein (LDL), and glycosylated hemoglobin (HbA1C) (*p* < 0.01).

**Table 1:** Descriptive statistics and non-parametric multinomial test for multiple levels of metabolic syndrome in health examination data.

**Factors**	**Health (0/5)**	**Met (1/5)**	**Met (2/5)**	**MetS (3/5)**	**MetS (4/5)**	**MetS (5/5)**	***p*-value**
	**n_0_ = 477**	**n_1_ = 295**	**n_2_ = 200**	**n_3_ = 102**	**n_4_ = 42**	**n_5_ = 13**	
	**No. (%)**	**No. (%)**	**No. (%)**	**No. (%)**	**No. (%)**	**No. (%)**	
CKD							
No	279 (58.5%)	148 (50.2%)	84 (42%)	39 (38.2%)	16 (38.1%)	4 (30.8%)	<0.001
Yes	198 (41.5%)	147 (49.8%)	116 (58%)	63 (61.8%)	26 (61.9%)	9 (69.2%)	
Sex							
Female	296 (62.1%)	126 (42.7%)	64 (32%)	26 (25.5%)	6 (14.3%)	1 (7.7%)	<0.001
Male	181 (37.9%)	169 (57.3%)	136 (68%)	76 (74.5%)	36 (85.7%)	12 (92.3%)	
**MEDIAN (IQR)**							
Age	42 (36–48)	45 (37–51)	45 (40–52)	45 (40–52)	45 (39–51)	44 (40–50)	<0.001
BMI	21.5 (19.9–23.2)	23.9 (22.3–25.9)	25 (23.3–27.5)	26.8 (24.9–29.8)	28.2 (26.6–30.9)	28.8 (25.8–31.7)	<0.001
Cholesterol	182 (163–202)	193 (170–213)	195 (172–219)	195 (166–214)	190 (158–215)	190 (139–241)	<0.001
LDL	112 (95–132)	128 (107–149)	134 (114–155)	134 (107–155)	124 (90–158)	135 (86–173)	<0.001
HbA1C	5.3 (5.1–5.4)	5.4 (5.2–5.6)	5.5 (5.3–5.7)	5.6 (5.3–5.9)	5.7 (5.4–6.0)	6.5 (6.0–7.3)	<0.001
GOT	19 (16–23)	20 (17–25)	21 (18–26)	23 (18–30)	26 (21–35)	26 (22–52)	<0.001
GPT	16 (12–22)	21 (15–31)	25 (17–35)	30 (20–47)	40.5 (24–58)	43 (23–99)	<0.001
γGT	13 (10–19)	18 (13–27)	22 (17–36)	25 (18–42)	35 (26–55)	37 (23–74)	<0.001
T_bilirubin	0.6 (0.4–0.8)	0.6 (0.5–0.8)	0.7 (0.4–0.9)	0.65 (0.5–1.0)	0.6 (0.4–0.9)	0.8 (0.5–1.2)	0.221
ALKp	55 (46–65)	58 (49–69)	62 (53–74)	61 (53–71)	67 (58–79)	59 (52–70)	<0.001
AFP	2.21 (1.56–3.02)	2.15 (1.57–3.2)	2.36 (1.72–3.24)	2.31 (1.62–3.07)	2.31 (1.66–3.15)	2.83 (2.28–4.70)	0.068
E score	3.9(3.3–4.6)	4.0 (3.4–4.7)	4.3 (3.5–5.1)	4.9 (4.0–5.5)	5.1 (4.4–6.8)	6.1 (4.6–6.8)	<0.001
CAP score	221(197–248)	250 (217–281)	272 (242–310)	298 (251–331)	327 (296.5–359)	323 (276–370)	<0.001
Albumin	4.6 (4.4–4.7)	4.6 (4.4–4.8)	4.6 (4.4–4.8)	4.6 (4.5–4.8)	4.6 (4.4–4.9)	4.8 (4.5–5.0)	0.007
BUN	12 (10–14)	12 (10–14)	12 (10–15)	12 (10–15)	13 (11–16)	12 (11–15)	0.009
Creatinine	0.7 (0.6–0.9)	0.8 (0.6–0.9)	0.8 (0.7–1.0)	0.9 (0.7–1.0)	0.9 (0.8–1.0)	1.0 (0.8–1.1)	<0.001
UA	4.8 (4.1–5.9)	5.5 (4.6–6.7)	6.0 (5.2–7.1)	6.3 (5.4–7.1)	6.9 (5.8–7.8)	7.1 (6.7–7.9)	<0.001
TSH	1.87 (1.24–2.61)	1.83 (1.30–2.48)	1.91 (1.24–2.54)	1.74 (1.25–2.45)	1.82 (1.12–2.75)	2.12 (1.39–2.65)	0.971

**Table 2:** Multinomial stepwise logistic regression analysis of risk factors related to metabolic syndrome.

**Factor**	**Met (1/5)**		**Met (2/5)**		**MetS (3/5)**		**MetS (4/5)**		**MetS (5/5)**		**Likelihood**
	**n_1_ = 295**		**n_2_ = 200**		**n_3_ = 102**		**n_4_ = 42**		**n_5_ = 13**		**Ratio Test**
	**OR**	***p*-value**	**OR**	***p*-value**	**OR**	***p*-value**	**OR**	***p*-value**	**OR**	***p*-value**	***p*-value**
Age	1.011	0.295	1.037	0.003	1.038	0.016	1.047	0.037	1.055	0.132	**0.032**
BMI	**1.392**	<0.001	**1.525**	<0.001	**1.825**	<0.001	**1.795**	<0.001	**1.877**	<0.001	**<0.001**
γGT	1.025	<0.001	1.033	<0.001	1.027	0.001	1.035	<0.001	1.039	<0.001	**<0.001**
CAPscore	1.003	0.230	1.005	0.051	1.009	0.008	1.024	<0.001	1.017	0.027	**<0.001**
UA	0.930	0.277	1.064	0.430	1.035	0.734	**1.282**	0.067	**1.772**	0.005	**0.014**
Cholesterol	0.985	0.028	0.962	<0.001	0.983	0.154	0.990	0.490	0.975	0.304	**0.002**
LDL	1.029	<0.001	1.056	<0.001	1.030	0.017	1.016	0.301	1.034	0.189	**<0.001**
HbA1C	**1.559**	0.087	**3.264**	<0.001	**4.717**	<0.001	**4.403**	<0.001	**7.447**	<0.001	**<0.001**

*The baseline of multinomial logistic regression for the health group is (0/5) without any metabolic syndrome disorders. After stepwise regression, only eight factors were retained. High odds ratios are in bold*.

**Figure 2:**
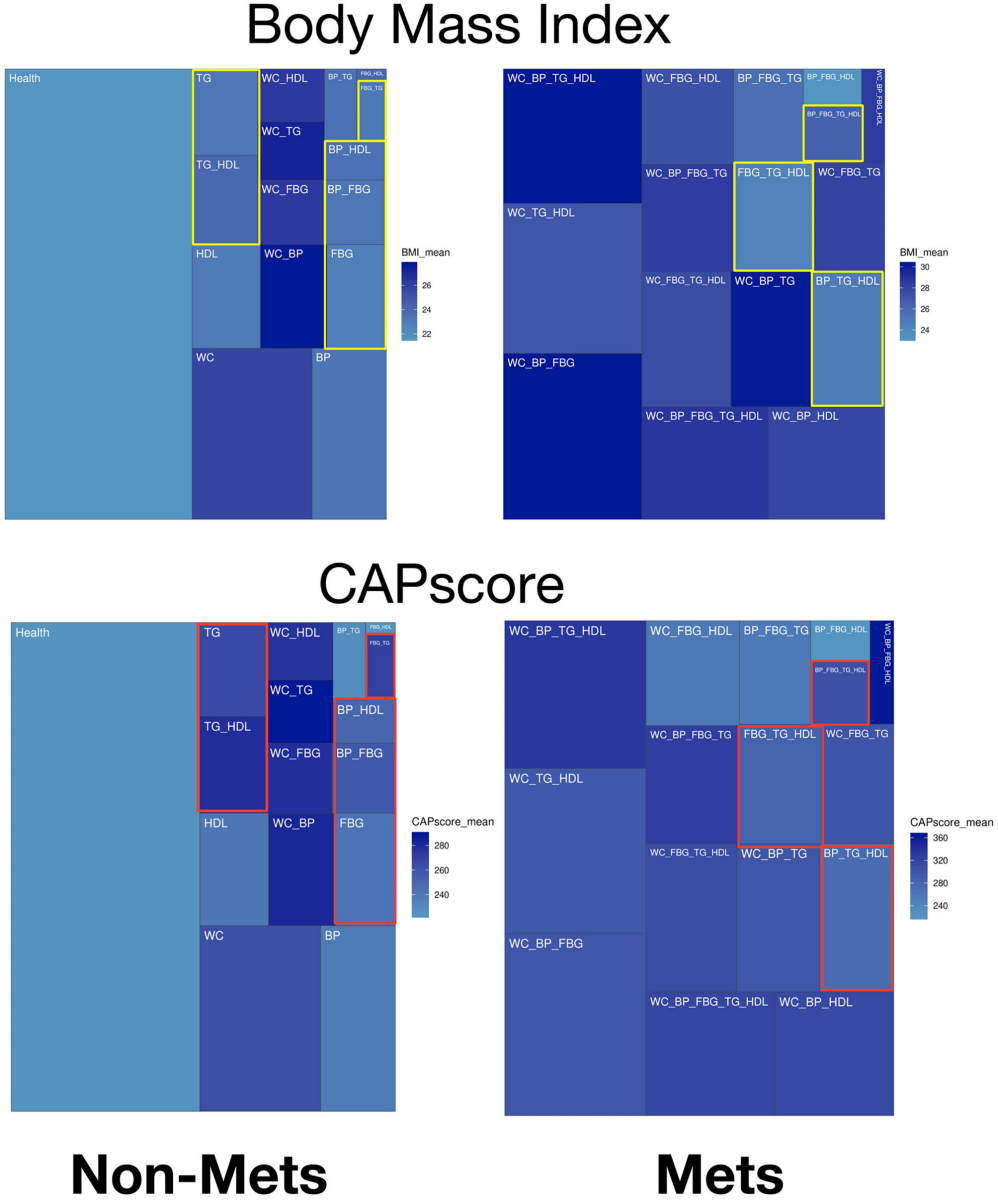
Treemaps of significant predictors within different combinations of metabolic components for non-MetS and MetS subjects. Body mass index (BMI) is the upper panel and CAP score is the lower panel.

In the treemaps presented in Figure 2 and Supplementary Figure A, gradient colors display specific patterns of significant predictors comparing groups with different numbers of metabolic components. The non-obese potential MetS populations are highlighted with color rectangles as comparison in treemaps. In Figure 2, the upper panel on BMI shows there is general positive correlation between BMI and waist circumference (WC). However, the highlighted yellow rectangles show some patients without elevated WC/ has low BMI, and yet many of these subjects have Mets. CAP score. Figure 2's lower panel on CAP score, shows the distribution of CAP score for different types of subjects. The highlighted red rectangles show the non-obese subjects, where the mean of CAP score is ~260.

**Figure 3:**
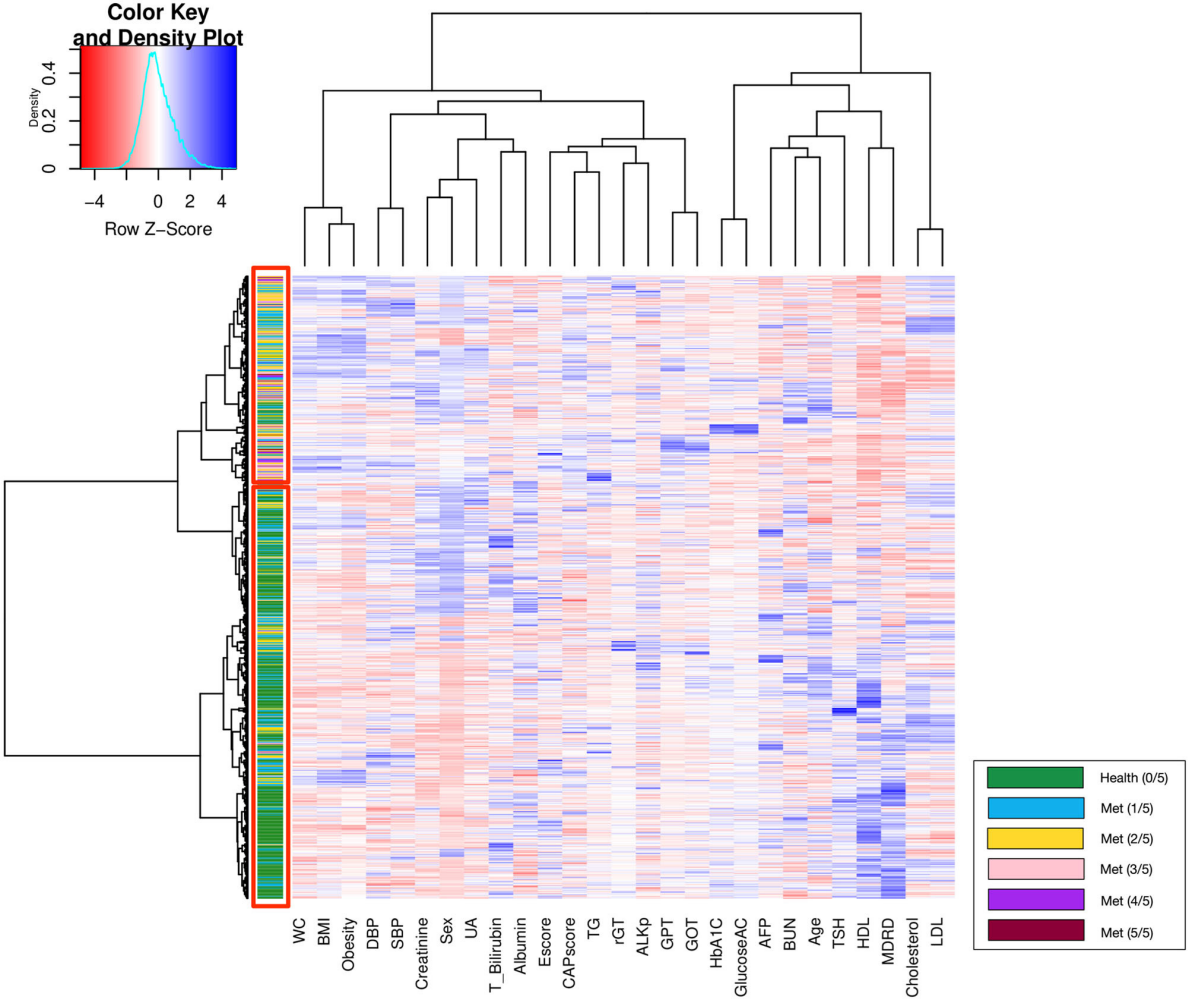
Heatmap for clustering patients according to the results from the medical records.

Unsupervised hierarchical clustering determined the similarity and classification between groups with different numbers of metabolic components; the corresponding heatmap is displayed in Figure 3. Patients with similar physiological records were clustered into the same group via hierarchical clustering analysis. In general, the upper red rectangle contains subjects with increased numbers of metabolic components, and the lower red rectangle contains healthy subjects (green), which do not have any Mets components. However, occasionally a few of the subjects do not follow the above described pattern.

**Figure 4:**
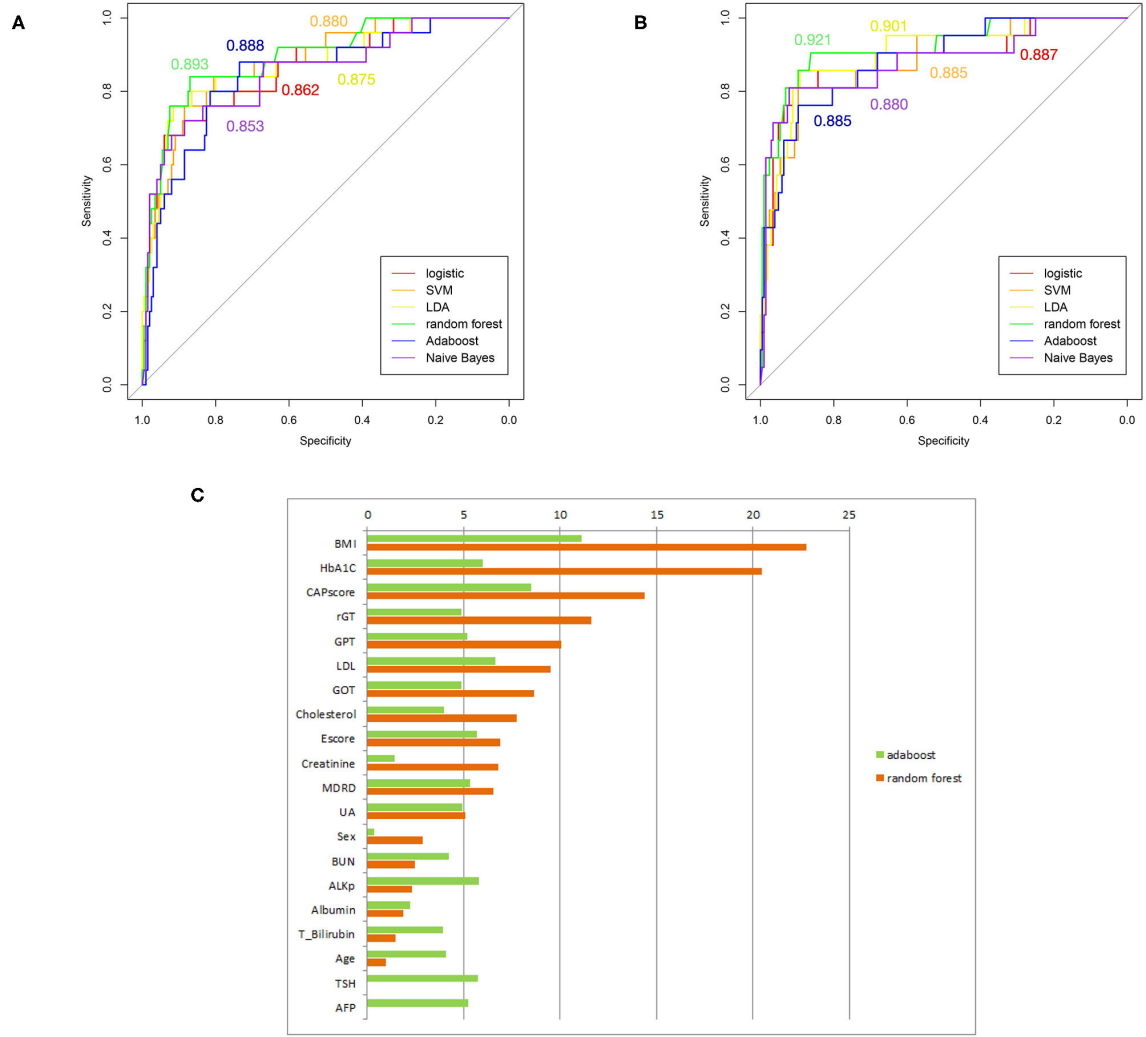
**(A)** ROC curves of several machine-learning models based on the comparison of ATP III criteria. **(B)** ROC curves of several machine-learning models based on the comparison of IDP criteria. **(C)** Ranking of predictors according to ensemble learning. The respective C-statistics for each model, are given according to the chosen color for the model.

Several supervised learning models were used to predict MetS according to both ATP III and International Diabetes Federation (IDF) criteria as the ground truth, and the performance of these models is illustrated in Figures 4A,B and Table 3. The rank of variable importance for ensemble learning summarization of multiple classifiers is represented in Figure 4C.

**Table 3:** Performance of different machine-learning models on predicting metabolic syndrome using ATP III, JIS, NHLBI, and IDF criteria.

**Model**	**Criteria**	**Accuracy**	**Sensitivity**	**Specificity**	**c–statistic**
Logistic	ATPIII	0.902	0.520	0.950	0.862
LDA	&	0.898	0.545	0.936	0.875
SVM	JIS	0.902	0.400	0.965	0.880
Random Forest	&	0.922	0.440	0.980	0.893
Adaboost	NHLBI	0.893	0.440	0.950	0.888
Naïve Bayes		0.853	0.720	0.870	0.853
Logistic	IDF	0.929	0.619	0.961	0.887
LDA		0.916	0.545	0.956	0.901
SVM		0.916	0.476	0.961	0.885
Random Forest		0.947	0.571	0.985	0.921
Adaboost		0.911	0.429	0.961	0.885
Naïve Bayes		0.893	0.810	0.902	0.880

*AUC, area under curve*.

**Figure 5:**
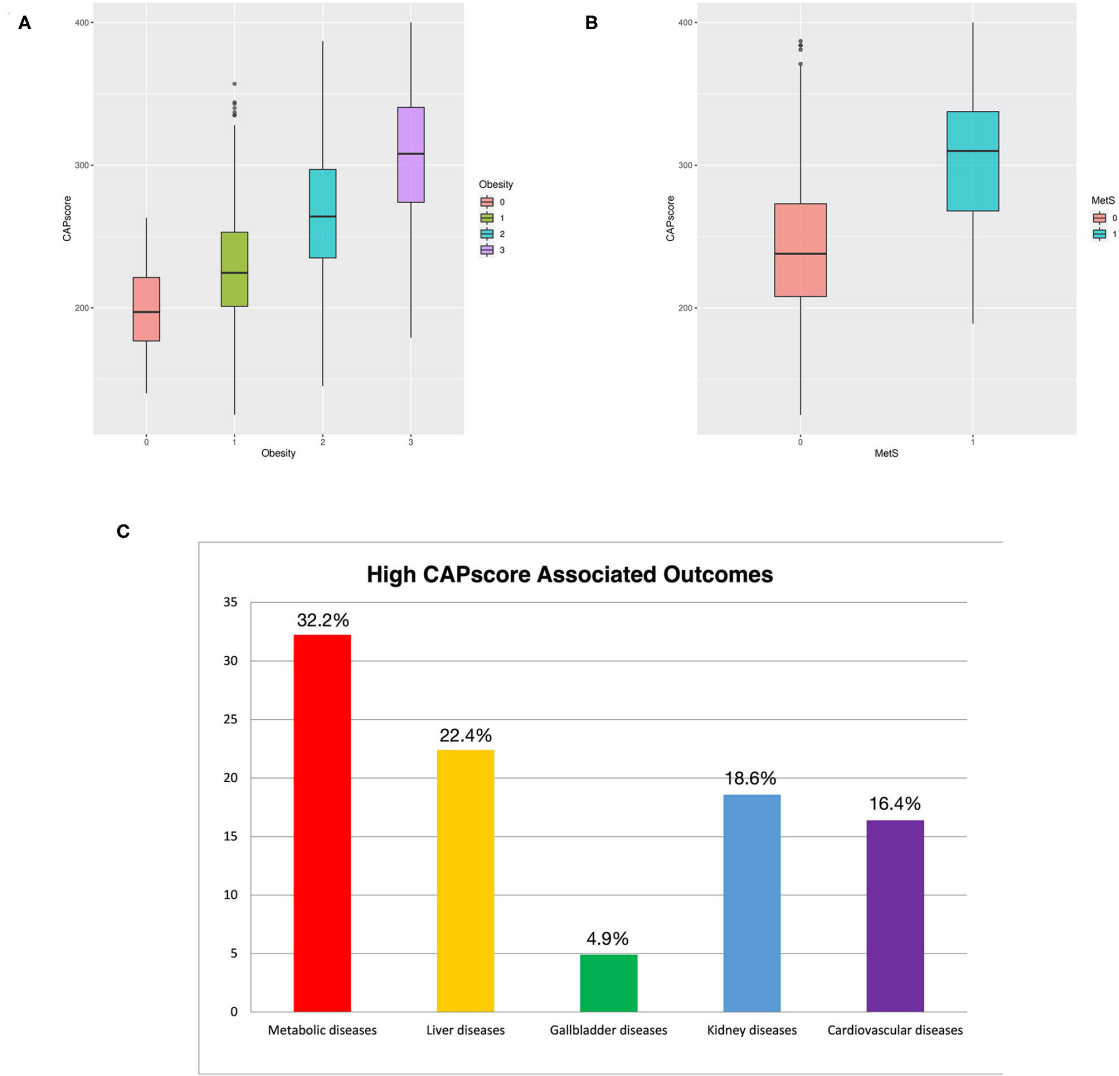
**(A)** Boxplot of CAP score comparing patients with and without obesity. **(B)** Boxplot of CAP score comparing patients with and without MetS. **(C)** Percentage of outcome diseases among patients with CAP score of higher more than 260. Patients that were loss during follow-up were deleted in the calculation of percentage.

The relationship between CAP score and obesity, as well as MetS, is shown in Figures 5A,B. The box plots presented in Figure 5B show that CAP score was positively related to MetS. Four attending physicians conduct an approximately 3 year follow-up of the patients with a CAP score higher than 260, and recorded metabolically associated diseases—including diabetes, cardiovascular disease, stroke, CKD, and dyslipidemia. The follow-up results are presented in Figure 5C, which shows that 32.2%, 22.4%, 18.6%, and 16.4% of the patients had metabolic diseases, liver-related diseases, kidney diseases, and cardiovascular diseases, respectively.

## Discussion

In statistical analysis, significant differences between groups with different numbers of metabolic components were discovered for several predictors. Because patients who have the same number of metabolic components may nonetheless have different combinations of the five components, their physical characteristics are diverse.

Furthermore, the classification of patients with different numbers of metabolic components that was visualized using clustering and a heat map revealed an overlapping representation at the left cluster label, although unsupervised machine learning made a strong contribution to the separation of the group with severe MetS (more than three components) and group with mild MetS (fewer than two components). Most patients with MetS were clustered in the upper portion, whereas healthier patients were clustered in the lower portion. Therefore, we applied several supervised learning models to predict MetS and found some representative predictors—such as CAP score, BMI, HbA1C, and γGT—that resulted in high accuracy and performance without any of the five criteria being involved in the models. Ensemble learning of random forest had highest performance in both ATP III and IDF criteria as ground truth with respective accuracy of 0.922 and 0.947 and c-statistic of 0.893 and 0.921; BMI with obesity, HbA1C, and CAP score were observed to be the best primary predictors for MetS (Figure 4C).

CAP score represents the severity of MetS because it reveals the extent of NAFLD (15, 42, 43). In previous research of several decision tree algorithms for MetS prediction, the threshold range of CAP score is also found to be approximately 290–300 (16). Similar to previous study, we found that if the goal is to identify both obese and non-obese patients, the cut off is ~290 (average of the 320 obese cut-off and 260 non-obese cut-off). Using the 260 non-obese CAP cut-off, we found that ~60% (43/72) of non-obese patients can be identified. This is likely because CAP is detecting NAFLD. In liver cells, NAFLD is caused by a considerable accumulation of triglycerides (44). Many evidence supports the connection between MetS and NAFLD. NAFLD is actually considered as the hepatic manifestation of MetS. Insulin resistance is the failure of cell to normally respond to insulin to reduce blood glucose level and is the key pathogenic feature of MetS. Insulin resistance is now identified as the most common risk factor for development and progression of NAFLD (45–47). In clinical laboratory examination, TG and FBG measurements are easily disturbed by many factors including incomplete fasting and medication. Therefore, CAP score measurements are more convenient and may be an alternative tool for detecting MetS, especially for the hard-to-detect non-obese patients.

The patients in the WC plus TG and WC plus BP metabolic component groups had higher BMI than those in the other groups (Figure 2). This implies that obesity is one of the leading risk factors for MetS (16). Moreover, multiple machine-learning models had high accuracy and performance for both the ATP III and IDF criteria. In particular, CAP score is also one of the primary variables in ensemble learning, giving machine-learning models high prediction ability (Figure 4C and Table 3). In addition, Figure 5A reveals that CAP score was proportional to degree of obesity. Fibroscan, a non-invasive method of screening for liver disease, is widely applied in detecting and treating NAFLD patients with MetS may be taken into consideration by experts and physicians.

Numerous cross-sectional and prospective studies have investigated the relationship between baseline γGT and the development of MetS (48–51). According our study, γGT is a valuable predictor of MetS because patients with TG and FBG metabolic components have elevated γGT (Supplementary Figure A). The non-obese metabolic health patients can be detected early to prevent progress of metabolic disorders to MetS. Moreover, the more glycemic level increases, the higher prevalence of NAFLD is (52). Several methods can evaluate the ranges of glycemia, containing HbA1c and FBG. HbA1c reflects the mean of glycaemia over the past 8–12 weeks and is applied to assess chronic glycemic level (53). Insulin resistance is a primary factor of NAFLD, and HbA1c correlates more strongly with insulin resistance than does FBG (54, 55).

A prominent relationship was illustrated between serum UA level and the risk for metabolic disorders in a meta-analysis of prospective studies. A linear relationship was speculated to exist between elevated UA and MetS/NAFLD incidence (56). Hyperuricemia is associated with histologically severe NAFLD (57). Furthermore, several research has identified UA as an independent risk factor for cardiometabolic diseases, indicating that UA can be regarded as a essential therapeutic target for patients with these diseases and particularly those with hyperuricemia (58).

This study has some limitations. First, the data only represent an Asian population; the CAP score cut-off at which fatty liver disease increases metabolic risks may vary for different races. Second, the data are collected from one HC and reflected the information of healthier population. Therefore, the bias in data distribution cannot be avoided. The more the information included on patients with severe MetS, the more robust is the distribution represented. Because of this limitation, this study focused on early intervention for patients to prevent the occurrence of MetS. Third, this is a single-center study involving self-paid health examination subjects that were prospectively follow-up in the same hospital, and only 40.4% of patients with CAP score >260 were successfully tracked in our hospital. A large number of patients with loss of follow-up implies that the metabolic-related risks may have been underestimated; therefore, the significance of fatty liver disease, measured using FibroScan, in MetS is probably higher than that determined in our study. In the future, it will be interesting to follow-up the medical record of these patients at other hospitals and apply machine learning in improving the prediction for cardiometabolic events for different types of Mets patients.

## Conclusion

Machine learning and big data visualization can depict the complicated relationships between metabolic components and potential risk factors. The potential MetS patients can be captured by machine learning for prevention especially for those non-obese population. In the future, more data on CAP scores from the healthy population and those with severe MetS should be collected to establish a more robust investigation. Moreover, analyzing data of different races could enhance the achievement of data visualization to describe the association between CAP score cut-off and MetS for different particular populations.

## Data Availability Statement

The datasets generated for this article are not publicly available due to the confidentiality concerns/ethical restriction but are available from the author on reasonable request. Requests to access the datasets should be directed to Shy-Shin Chang, sschang0529@gmail.com.

## Ethics Statement

The studies involving human participants were reviewed and approved by Taipei Medical University-Joint Institutional Review Board (TMU-JIRB No.: N202003088). Written informed consent for participation was not required for this study in accordance with the national legislation and the institutional requirements.

## Author Contributions

C-SY and S-SC: study conception and design, analysis and interpretation of data, and acquisition of funding. C-SY, S-SC, R-JC, and JLW: acquisition of data. R-JC, S-SC, C-HL, and Y-JL: medical insight consultation. C-SY and R-JC: intelligence insight consultation. R-JC, C-SY, and S-SC: drafting of the manuscript. All authors contributed to the article and approved the submitted version.

## Conflict of Interest

The authors declare that the research was conducted in the absence of any commercial or financial relationships that could be construed as a potential conflict of interest.
