# Supplementary material for: Identify the Characteristics of Metabolic Syndrome and Non-obese Phenotype: Data Visualization and a Machine Learning Approach
Source: Front Med (Lausanne). 2021 Apr 7;8:626580. doi: 10.3389/fmed.2021.626580 (PMC8058220; doi:10.3389/fmed.2021.626580)
Supplement: Supplementary file 1 [file Table_1.DOCX]

**Supplementary Table 1.** A comparison of the different criteria for Mets.

|  | Modified ATP III Guidelines* Criteria | IDF Criteria | JIS Criteria | NHLBI Criteria |
| --- | --- | --- | --- | --- |
| Definition | If any three of the following five criteria are present, then the person is identified as having MetS. | To be defined as having MetS, a person must have abdominal obesity** plus any two of other four factors. | If any three of the following five criteria are present, then the person is identified as having MetS. | If any three of the following five criteria are present, then the person is identified as having MetS. |
| Abdominal obesity |  |  |  |  |
| WC, cm |  |  |  |  |
| Male | ≧90 | ≧90 (Asian) | ≧90 (Asian) | ≧90 (Asian American) |
| Female | ≧80 | ≧80 (Asian) | ≧80 (Asian) | ≧80 (Asian American) |
|  |  |  | * IDF cut points is recommended to be used for non-Europeans |  |
| TG, mg/dL | ≥150 | ≥150 | ≥150 | ≥150 |
|  | Or receiving treatment for this lipid abnormality | Or receiving specific treatment for this lipid abnormality | Or taking drug treatment for elevated triglycerides | Or taking drug treatment for elevated triglycerides |
| BP, mmHg |  |  |  |  |
| Systolic | ≥130 | ≥130 | ≥130 | ≥130 |
| Diastolic | ≥85 | ≥85 | ≥85 | ≥85 |
|  | Or receiving treatment for their condition | Or treatment of previously diagnosed hypertension | Or have history of hypertension and taking antihypertensive drug treatment | Or have history of hypertension and taking antihypertensive drug treatment |
| HDL |  |  |  |  |
| cholesterol, mg/dL |  |  |  |  |
| Male | <40 | <40 | <40 | <40 |
| Female | <50 | <50 | <50 | <50 |
|  | Or receiving treatment for this lipid abnormality | Or receiving specific treatment for this lipid abnormality | Or taking drug treatment for reduced HDL-C | Or taking drug treatment for reduced HDL-C |
| FBG, mg/dL | ≥100 | ≥100 | ≥100 | ≥100 |
|  | Or receiving drug treatment for elevated glucose | Or previously diagnosed type 2 diabetes | Or taking drug treatment for elevated glucose | Or taking drug treatment for elevated glucose |

*Modified by Health Promotion Administration, Ministry of Health and Welfare, Taiwan.

** If BMI is >30 kg/m^2^, then it can be assumed as abdominal obesity without measuring waist circumference.
