# Supplementary material for: Identify the Characteristics of Metabolic Syndrome and Non-obese Phenotype: Data Visualization and a Machine Learning Approach
Source: Front Med (Lausanne). 2021 Apr 7;8:626580. doi: 10.3389/fmed.2021.626580 (PMC8058220; doi:10.3389/fmed.2021.626580)
Supplement: Supplementary file 2 [file Table_2.docx]

**Supplementary Table 2.** Procedure of physical examination and health assessment

| 1. Front desk check in and receiving a radio-frequency identification (RFID) watch | 2. Health examination procedure explanation, identity confirmation via RFID watch, and pre-examination preparation confirmation (i.e., fasting for 8 hours before appointment) |
| --- | --- |
| 3. Body measurement stage 1 | 4. Body measurement stage 2 |
| 5. Blood and other specimen collection | 6. Ultrasonic examination |
| 7. Fibroscan examination 1 | 8. Fibroscan examination 2 |
| 9. Medical Consultation and AI Health Care APP introduction | 10. End of the examination and identity confirmation |
