# Supplementary material for: Identify the Characteristics of Metabolic Syndrome and Non-obese Phenotype: Data Visualization and a Machine Learning Approach
Source: Front Med (Lausanne). 2021 Apr 7;8:626580. doi: 10.3389/fmed.2021.626580 (PMC8058220; doi:10.3389/fmed.2021.626580)
Supplement: Supplementary file 3 [file Table_3.docx]

**Supplementary Table 3.** List of input variables for machine learning.

| **Anthropometric** variables | Unit |
| --- | --- |
| Sex | M/F |
| Age | year |
| Waist circumference (WC)* | cm |
| Body mass index (BMI) | kg/m^2^ |
| Systolic blood pressure* | mmHG |
| Diastolic blood pressure* | mmHG |
| **Electronic health care records** variables |  |
| Fasting blood glucose (FBG)* | mg/dL |
| Triglycerides (TG)* | mg/dL |
| High-density lipoprotein (HDL)* | mg/dL |
| Low-density lipoprotein (LDL) | mg/dL |
| Cholesterol | mg/dL |
| Hemoglobin A1c (HbA1C) | % |
| Glutamic-oxalocetic transaminase (GOT) | IU/L |
| Glutamate pyruvate transaminase (GPT) | IU/L |
| γ-Glutamyl transpeptidase (γGT) | U/L |
| Total bilirubin (T_bilirubin) | mg/dL |
| Alkaline phosphatase (ALKp) | IU/L |
| Alpha-fetoprotein (AFP) | ng/mL |
| Albumin | g/dL |
| Blood urea nitrogen (BUN) | mg/dL |
| Creatinine | mg/dL |
| Uric acid (UA) | mg/dL |
| Thyroid-stimulating hormone (TSH) | μIU/mL |
| **Fibroscan** free-text variables |  |
| E score | kPa |
| CAP score | dB/m |

*Metabolic syndrome is a cluster of disorders, including insulin resistance or hyperglycemia, visceral adiposity (identified by a large waistline or overweight), atherogenic dyslipidemia (e.g., raised TG or reduced high-density lipoprotein), and endothelial dysfunction (characterized by elevated BP).
