# Supplementary material for: Identify the Characteristics of Metabolic Syndrome and Non-obese Phenotype: Data Visualization and a Machine Learning Approach
Source: Front Med (Lausanne). 2021 Apr 7;8:626580. doi: 10.3389/fmed.2021.626580 (PMC8058220; doi:10.3389/fmed.2021.626580)
Supplement: Supplementary file 4 [file Table_4.docx]

**Supplementary Table 4.** Definition of obesity in Taiwan for Asian population

| Body Mass Index (BMI) Categories | Code | BMI (kg/m^2^) |
| --- | --- | --- |
| Underweight | 0 | <18.5 |
| Normal | 1 | 18.5–24 |
| Overweight | 2 | 24–27 |
| Obesity | 3 | ≥27 |
