# Supplementary material for: Identify the Characteristics of Metabolic Syndrome and Non-obese Phenotype: Data Visualization and a Machine Learning Approach
Source: Front Med (Lausanne). 2021 Apr 7;8:626580. doi: 10.3389/fmed.2021.626580 (PMC8058220; doi:10.3389/fmed.2021.626580)
Supplement: Supplementary file 5 [file Table_5.docx]

**Supplementary Table 5.** List of packages and its function about machine learning models in this study

| Models | types | Library Package and Code |
| --- | --- | --- |
| Logistic | Regression | library(regclass)  library(aod)  library(ggplot2)  glm(formula, family = gaussian, data, weights, subset,  na.action, start = NULL, etastart, mustart, offset,  control = list(...), model = TRUE, method = "glm.fit",  x = FALSE, y = TRUE, singular.ok = TRUE, contrasts = NULL, ...) |
| LDA | Supervised | library(MASS)  lda(formula, data, ..., subset, na.action) |
| SVM | Supervised | library (e1071)  library(kernlab)  ## S3 method for class 'formula'  svm(formula, data = NULL, ..., subset, na.action =  na.omit, scale = TRUE) |
| Random Forest | Supervised | library(randomForest)  ## Default S3 method:  randomForest(x, y=NULL, xtest=NULL, ytest=NULL, ntree=500,mtry=if (!is.null(y) && !is.factor(y)) max(floor(ncol(x)/3), 1) else floor(sqrt(ncol(x))), replace=TRUE, classwt=NULL, cutoff, strata, sampsize = if (replace) nrow(x) else ceiling(.632*nrow(x)), nodesize = if (!is.null(y) && !is.factor(y)) 5 else 1, maxnodes = NULL, importance=FALSE, localImp=FALSE, nPerm=1, proximity, oob.prox=proximity, norm.votes=TRUE, do.trace=FALSE, keep.forest=!is.null(y) && is.null(xtest), corr.bias=FALSE, keep.inbag=FALSE, ...) |
| Adaboost | Supervised | library(adabag)  library(rpart)  boosting(formula, data, boos = TRUE, mfinal = 100, coeflearn = 'Breiman', control,...) |
| Naïve Bayes | Supervised | library(e1071)  naiveBayes(formula, data, laplace = 0, ..., subset, na.action = na.pass) |
| Heatmap | Unsupervised | library(RColorBrewer)  library(gplots)  require(made4)  heatmap.2 (x,  # dendrogram control  Rowv = TRUE,  Colv=if(symm)"Rowv" else TRUE,  distfun = dist,  hclustfun = hclust,  dendrogram = c("both","row","column","none"),  reorderfun = function(d, w) reorder(d, w),  symm = FALSE,  # data scaling  scale = c("none","row", "column"),  na.rm=TRUE,  # image plot  revC = identical(Colv, "Rowv"),  add.expr,  # mapping data to colors  breaks,  symbreaks=any(x < 0, na.rm=TRUE) \|\| scale!="none",  # colors  col="heat.colors",  # block sepration  colsep,  rowsep,  sepcolor="white",  sepwidth=c(0.05,0.05),  # cell labeling  cellnote,  notecex=1.0,  notecol="cyan",  na.color=par("bg"),  # level trace  trace=c("column","row","both","none"),  tracecol="cyan",  hline=median(breaks),  vline=median(breaks),  linecol=tracecol,  # Row/Column Labeling  margins = c(5, 5),  ColSideColors,  RowSideColors,  cexRow = 0.2 + 1/log10(nr),  cexCol = 0.2 + 1/log10(nc),  labRow = NULL,  labCol = NULL,  srtRow = NULL,  srtCol = NULL,  adjRow = c(0,NA),  adjCol = c(NA,0),  offsetRow = 0.5,  offsetCol = 0.5,  colRow = NULL,  colCol = NULL,  # color key + density info  key = TRUE,  keysize = 1.5,  density.info=c("histogram","density","none"),  denscol=tracecol,  symkey = any(x < 0, na.rm=TRUE) \|\| symbreaks,  densadj = 0.25,  key.title = NULL,  key.xlab = NULL,  key.ylab = NULL,  key.xtickfun = NULL,  key.ytickfun = NULL,  key.par=list(),  # plot labels  main = NULL,  xlab = NULL,  ylab = NULL,  # plot layout  lmat = NULL,  lhei = NULL,  lwid = NULL,  # extras  extrafun=NULL,  ...  ) |
| Treemap | Data Visualization | library(ggplot2)  library(treemapify)  treemapify(  data,  area,  subgroup,  subgroup2,  subgroup3,  layout = "squarified",  start = "bottomleft",  fill = NULL,  label = NULL,  group = NULL,  fixed = NULL,  xlim = c(0, 1),  ylim = c(0, 1)  ) |
