# Supplementary material for: Identify the Characteristics of Metabolic Syndrome and Non-obese Phenotype: Data Visualization and a Machine Learning Approach
Source: Front Med (Lausanne). 2021 Apr 7;8:626580. doi: 10.3389/fmed.2021.626580 (PMC8058220; doi:10.3389/fmed.2021.626580)
Supplement: Supplementary file 6 [file Table_6.docx]

**Supplementary Table 6.** List of combinations in metabolic syndrome disorders

| Set | Code | Combination |
| --- | --- | --- |
| Health (0/5) | 0 | No disorders in metabolic syndrome involved |
| MetS (1/5) | 1 | WC |
|  | 2 | BP |
|  | 3 | FBG |
|  | 4 | TG |
|  | 5 | HDL |
| MetS (2/5) | 6 | WC; BP |
|  | 7 | WC; FBG |
|  | 8 | WC; TG |
|  | 9 | WC; HDL |
|  | 10 | BP; FBG |
|  | 11 | BP; TG |
|  | 12 | BP; HDL |
|  | 13 | FBG; TG |
|  | 14 | FBG; HDL |
|  | 15 | TG; HDL |
| MetS(3/5) | 1 | WC; BP; FBG |
|  | 2 | WC; BP; TG |
|  | 3 | WC; BP; HDL |
|  | 4 | WC; FBG; TG |
|  | 5 | WC; FBG; HDL |
|  | 6 | WC; TG; HDL |
|  | 7 | BP; FBG; TG |
|  | 8 | BP; FBG; HDL |
|  | 9 | BP; TG; HDL |
|  | 10 | FBG; TG; HDL |
| MetS(4/5) | 11 | WC; BP; FBG; TG |
|  | 12 | WC; BP; FBG; HDL |
|  | 13 | WC; BP; TG; HDL |
|  | 14 | WC; FBG; TG; HDL |
|  | 15 | BP; FBG; TG; HDL |
| MetS(5/5) | 16 | WC; BP; FBG; TG; HDL |
