# Supplementary material for: Identify the Characteristics of Metabolic Syndrome and Non-obese Phenotype: Data Visualization and a Machine Learning Approach
Source: Front Med (Lausanne). 2021 Apr 7;8:626580. doi: 10.3389/fmed.2021.626580 (PMC8058220; doi:10.3389/fmed.2021.626580)
Supplement: Supplementary file 7 [file Image_1.pdf]

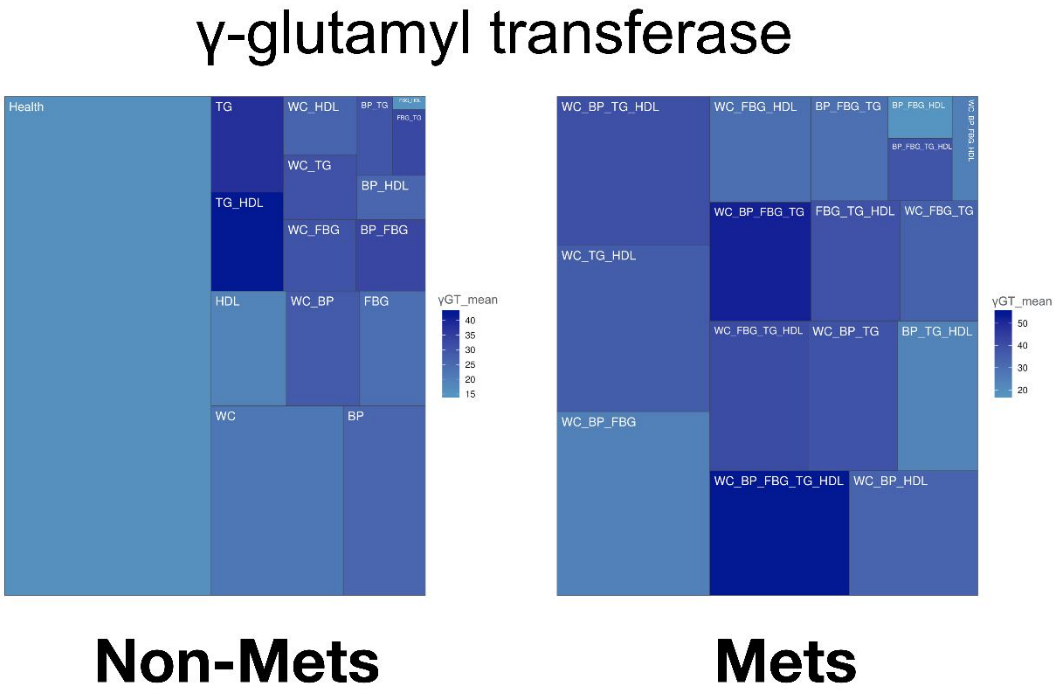

Supplementary Figure A.1 Treemap of γGT within different combinations of metabolic components for MetS.

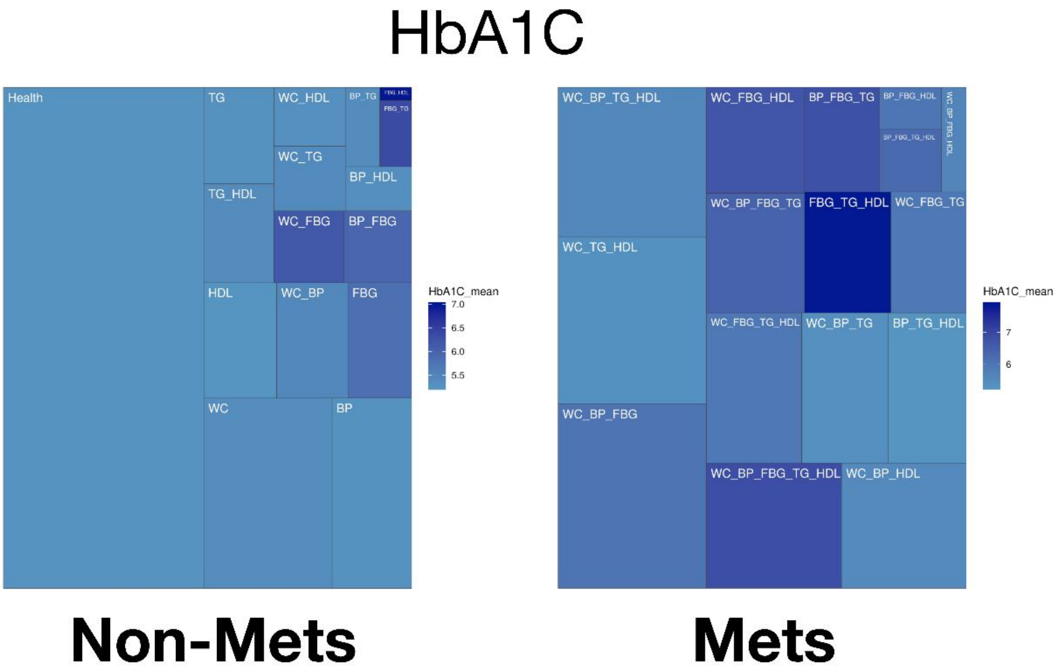

Supplementary Figure A.2 Treemaps of significant predictors within different combinations of metabolic components for MetS.

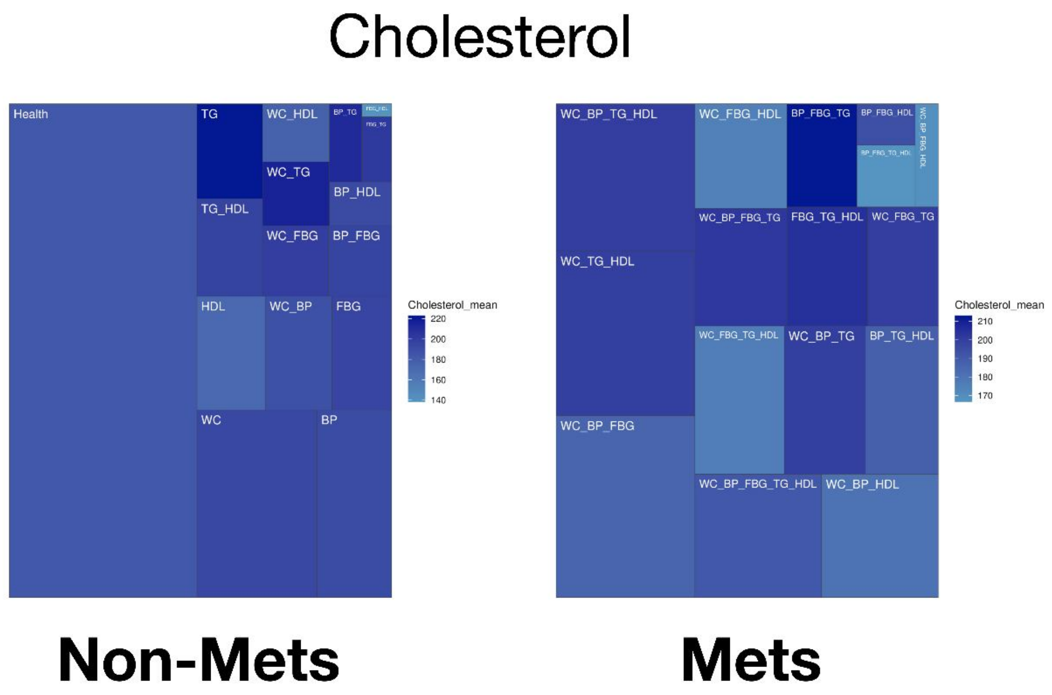

Supplementary Figure A.3 Treemaps of significant predictors within different combinations of metabolic components for MetS.

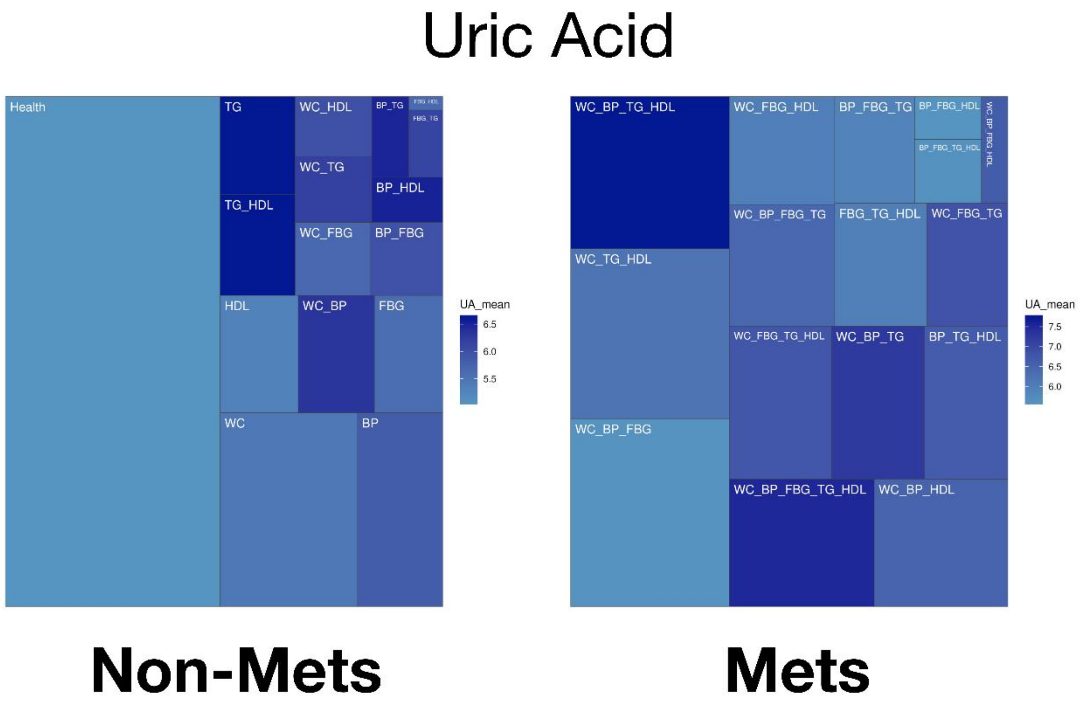

Supplementary Figure A.4 Treemaps of significant predictors within different combinations of metabolic components for MetS.
